# Progress in Titanium Metal Powder Injection Molding

**DOI:** 10.3390/ma6083641

**Published:** 2013-08-20

**Authors:** Randall M. German

**Affiliations:** Mechanical Engineering, San Diego State University, 5500 Campanile Drive, San Diego, CA 92128, USA; E-Mail: rgerman@mail.sdsu.edu; Tel.: +1-858-922-4985

**Keywords:** titanium, metal powder injection molding, purity, density, sintering, microstructure, alloying, powder characteristics, particle size, oxygen control

## Abstract

Metal powder injection molding is a shaping technology that has achieved solid scientific underpinnings. It is from this science base that recent progress has occurred in titanium powder injection molding. Much of the progress awaited development of the required particles with specific characteristics of particle size, particle shape, and purity. The production of titanium components by injection molding is stabilized by a good understanding of how each process variable impacts density and impurity level. As summarized here, recent research has isolated the four critical success factors in titanium metal powder injection molding (Ti-MIM) that must be simultaneously satisfied—density, purity, alloying, and microstructure. The critical role of density and impurities, and the inability to remove impurities with sintering, compels attention to starting Ti-MIM with high quality alloy powders. This article addresses the four critical success factors to rationalize Ti-MIM processing conditions to the requirements for demanding applications in aerospace and medical fields. Based on extensive research, a baseline process is identified and reported here with attention to linking mechanical properties to the four critical success factors.

## 1. Introduction

Titanium component production by the metal powder injection molding process (Ti-MIM) was the subject of much effort over the past few decades with several reports showing the incremental steps in powders, binders, debinding, sintering, and post-sintering details [1,2,3,4,5,6,7,8,9,10,11,12,13,14,15,16,17,18,19,20,21,22,23,24,25,26,27,28,29,30,31,32,33,34,35,36,37,38,39,40,41,42,43,44,45,46,47,48,49,50,51,52,53,54,55,56,57,58,59,60,61,62,63,64,65,66,67,68,69,70,71,72,73,74,75,76,77,78,79,80,81,82,83,84,85,86,87,88,89,90,91,92,93,94,95,96,97,98,99,100,101,102,103,104,105,106,107,108,109,110,111,112,113,114,115,116,117,118,119,120,121,122,123,124,125,126,127,128,129]. The intent is not to detail these studies, but to highlight the significant changes the titanium research has delivered. The progress was accelerated by novel powder synthesis routes delivering a variety of small powders that exhibited the required sintering shrinkage. With the major advances in powder fabrication Ti-MIM settled on −45 µm spherical particles to balance sintering densification (smaller particles are desired), impurity accumulation (large particles are desired), and component shape retention (small particles are required). With the recent surge in powder production, many development efforts arose in the injection molding research to evaluate how powders characteristics impact the rheology and sintering. Most importantly, progress in powder atomization provides the infrastructure platform to enable successful Ti-MIM. Accordingly, much effort is applied to form high purity titanium components using the Ti-MIM process [114,115,116,117,118,119,120,121,122,123,124,125,126,127,128,129,130,131,132,133,134,135,136,137,138,139,140,141,142,143].

In retrospect, powder synthesis had to move forward as a basis for enabling Ti-MIM’s projection into demanding applications. Currently, depending on powder and process quality, the applications are in three general segments:

(1) **Decorative** items where mechanical and other properties are not demanding, such as in watch cases;
(2) **Mechanical** components where mechanical and corrosion properties must exceed that of a stainless steel such as in medical surgical tools;
(3) **Life Critical** applications where titanium is needed for success such as in biomedical implants.

Attention to the powder quality and process details leads to three tiers for the powder-process technology. For example, decorative items are associated with a marketing advantage as evident when Ti-MIM sunglass frames emerged using hydride-dehydride powders. Several successes occurred with mechanical titanium components, including cell phone and firearm components by MIM. Now Ti-MIM is moving into taxing applications for biomedical implants and aerospace components. Research accompanying this effort carefully links the properties to powder-process decisions to document the optimal process. Later in this article the critical factors are tabulated to provide a sense on the current process evolution.

## 2. Background

Four parameters dominate the mechanical properties of sintered titanium—density, interstitial content, alloying, and microstructure. Usually the corrosion and biomedical attributes are likewise contingent on the same factors. Together, the optimal properties arise from Ti-MIM when it is sintered to a high density, with little contamination (largely oxidation), with alloying (such as Nb), and consolidated under conditions that avoid microstructure coarsening and defects such as inclusions.

**Figure 1:**
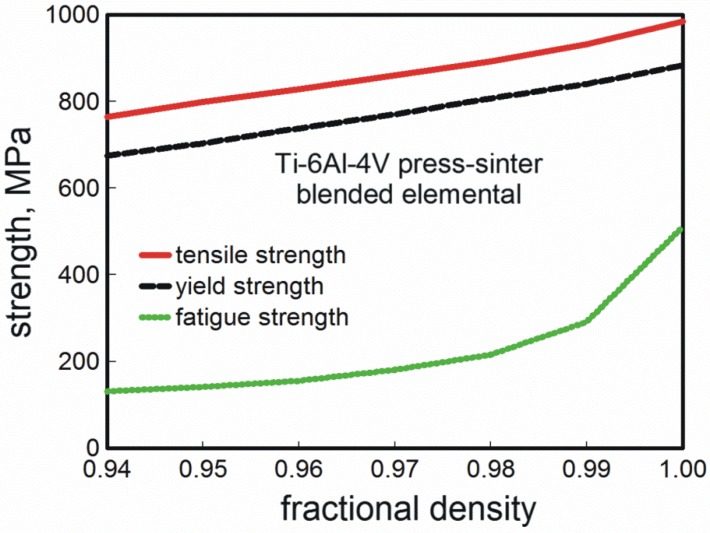
A plot of the mechanical properties for sintered Ti-6Al-4V from mixed powders *versus* fractional density showing the sensitivity of fatigue behavior as compared to tensile and yield strength.

The first point with respect to Ti-MIM is density. Residual pores degrade mechanical properties, so full density is desirable [114]. Further, ductility is sensitive to the test geometry [125]; round bars give a higher ductility. Likewise, surface porosity degrades properties, so some studies employ shot peening to improve fatigue strength by 100 MPa; this is because gas generates large pores in the sintered body, with pore sizes reaching up to 80 µm [124]. Indeed fracture toughness and fatigue strength are mechanical properties most sensitive to residual porosity, more so than tensile strength. As an example, fatigue strength jumps 18% with the elimination of the last 2% porosity by HIP [126]. Containerless hot isostatic pressing is a common means to attain full density after sintering. A classic demonstration for titanium is given in Figure 1 where tensile strength, yield strength, and fatigue strength are plotted for Ti-6Al-4V. Gains are evident with densification; in this plot the yield strength changes 30% as density increases from 94% to 100%, but fatigue strength increases 400%.

The second point with respect to Ti-MIM is the interstitial content. Interstitial oxygen, carbon, nitrogen, or hydrogen increase yield strength, tensile strength, and hardness, but decrease ductility. For sintered titanium, oxygen is the focus, leading to a sorting of alloy grades based on the oxygen content. Unalloyed commercially pure grade-1 titanium (CP Ti) must have below 1800 ppm oxygen, resulting in a tensile strength of 240 MPa with 24% fracture elongation. At higher oxygen levels, such as for grade-4 titanium, the oxygen ranges to 4000 ppm with tensile strength exceeding 550 MPa, but the ductility declines to 15% elongation. The control of interstitials is the largest difficulty with sintered titanium. This is because the impurities are soluble at the sintering temperature and there are no effective reducing agents. Thus contamination arriving with the powder is increased by furnace and substrate sources. Since oxygen is a major concern it is common to denote an oxygen equivalent impurity level, where each impurity is assigned a weighting factor with respect to property changes when compared to oxygen. Strength increases linearly with the oxygen equivalent [126].

The third point with respect to Ti-MIM is alloying. The compositions are copies of wrought alloys. Several alternative compositions exist, but for Ti-MIM the focus is on three alloys CP Ti, Ti-6Al-4V, and Ti-6Al-7Nb. By far, the most common alloy is Ti-6Al-4V. When taken to full density with less than 2000 ppm oxygen this alloy delivers tensile strength between 710 and 850 MPa with 12% elongation. Alloying with boron is advocated to control microstructure during sintering [122], but this is now outside the composition “equivalent” window and must be newly qualified for each application.

The sintered tensile strength varies with both the powder choice and processing details and generally is below the equivalent wrought level of 950 MPa and 14% elongation. This reflects a coarse grained microstructure concomitant with the time-temperature combinations required for sintering. Although a few alloys have been developed just for Ti-MIM to compensate for the processing sensitivities, as yet they have been put into production.

A final factor is the microstructure, pivoting on the grain size and mixture of phases after sintering. Microstructure coarsening during sintering tends to slightly reduce yield strength compared to wrought titanium. Accordingly, one option is to sinter to the closed-pore condition and rely on lower temperature hot isostatic pressing for final densification. This proves successful. When Ti-MIM performed with attention to the factors of powder, interstitial, density, alloying, and microstructure, the mechanical properties approach that attained in annealed wrought material, reaching about 975 MPa tensile strength and 14% elongation to fracture Unfortunately, without alloying and without optimal starting powder the optimized properties are lower at 483 MPa and 23% [138].The balance of this paper reviews the scientific steps needed to accomplish this success.

## 3. Ti-MIM Processing Optimization

The processing science for titanium traces to the early availability of titanium in a powder form. The first reports in the 1950s relied on spark sintering by Lenel [144]. This was followed by hot isostatic pressing [145]. The hot isostatic pressing (HIP) used inert handling to deliver low interstitial levels for high performance aerospace applications. Spherical, high purity and rather large rotating electrode powders played an important role, although now plasma atomized and even hydride-dehydride powders are widely used. Other efforts relied on lower cost powders, such as sponge fines and blended elemental powders, in die compaction. The progress was documented by the 1980 conference proceedings [146]. The HIP Ti-6Al-4V product delivered a tensile strength of 975 MPa with 14% elongation, similar to wrought material. Post-consolidation heat treatments enabled strength-ductility manipulations ranging up to an 1130 MPa tensile strength with 9% elongation. Without HIP full density was not attained so the properties were lower. In the best case, sintered titanium reached 920 MPa with 11% elongation at 98% density for Ti-6Al-4V. However, the fatigue and fracture toughness were typically lower than the comparable wrought product. When subjected to shot peening, fatigue strength is about 410 MPa, but when subjected to HIP and shot peening it is closer to 485 MPa [126].

Using the early base from press-sinter titanium sintering, Ti-MIM was demonstrated in 1988 [2]. Early reports showed an impressive 1000 MPa tensile strength, but just 2% elongation. On this basis, early Ti-MIM reached production status for decorative applications as early as 1991. The most notable application was in running shoe spikes; Leroy Burrell posted 100 m dash time of 9.88 s using ASICS shoes with low ductility but high strength Ti-MIM spikes. Dental orthodontic bracket efforts started soon after and some of the trials included surgical tools, automotive shifter knobs, toy components including model railroad train wheels, and eyeglass frames.

**Figure 2:**
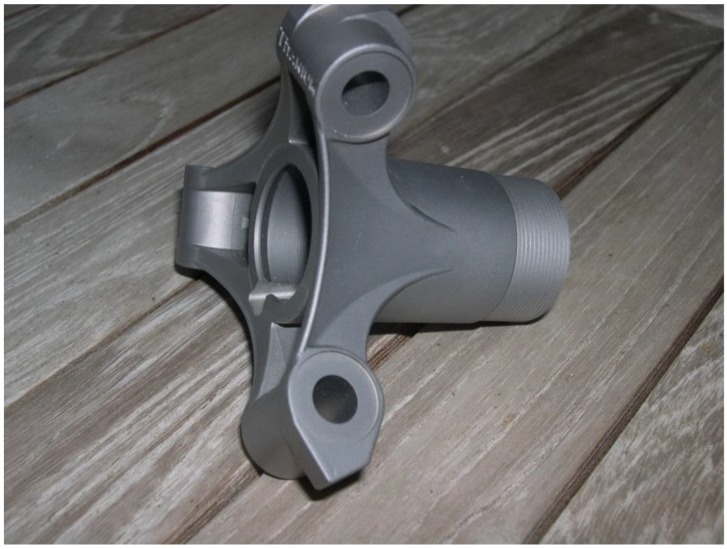
An example of a Ti-MIM component, in this case a tripod base.

Subsequently, scientific study refined processing with a primary focus on oxygen with a target of improved as-sintered ductility and corrosion resistance. Several detailed studies emerged disclosing the processing cycles required for titanium and its alloys, as reviewed in recent papers [114,115,116,117,118,119,120,121,122,123,124,125,126,127,128,129,130,131,132,133,134,135,136,137,138,139,140,141,142,143]. Along the way, in spite of many industry skeptics, Ti-MIM reached production status for several mechanical components. Several of these are shown in earlier design guides [98,103,125,136,139] and are not being repeated. One of the impressive Ti-MIM components is the tripod base shown in Figure 2.

Most recently Ti-MIM has penetrated life critical applications in dental, aerospace, medical, and chemical devices [115,116,120,121,122,123,124,125,126,127,128,129,134,135,140,141,142,143]. Based on success at research institutions, the high quality Ti-MIM process has been put into production with variants by several firms.

To achieve this status, Ti-MIM was a favorite research topic in the research community, in part because of the process sensitivity to so many factors. According, much effort is occurring to selectively piece together the best solutions. For example, El-Kadiri *et al.* [79] disclosed new titanium alloys using lower cost powder targeted at automotive applications. This work relied on −45 µm 99.8% pure sponge Ti powder alloyed using admixed Zr and Fe to form a liquid phase sintering system. A composition with 7.5% Fe and 5.0% Zr, sintered 1275 °C for 1 h gave 99% density with a high tensile strength but low ductility.

One study demonstrated a cold isostatic pressing step between debinding and sintering was favorable [113] while another found a polyacetal binder could be debound and sintered in a single step [72]. Miura *et al.* [31] produced Ti-6Al-7Nb using spherical gas atomized −325 mesh (−45 μm) Ti powder (0.008% C and 0.140% O) mixed with prealloyed Al-Nb, elemental Al and Nb, and Nb with prealloyed Ti-Al. For optimal properties, the powders were premixed, and then feedstock was mixed at 173 °C for 2.5 h to give 65 vol % solids loading. After molding, the wax phase was removed via heptane immersion for 6 h, followed by vacuum sweep-gas treatment at 430 °C. Sintering densification was at 1350 °C for 4 h. The sintered density was higher using prealloyed powders, exceeding 97% fractional density, with a tensile strength of 830 MPa and 11% elongation with 0.31% oxygen.

A wide range of issues are ahead in Ti-MIM. Use of mixed atomized and hydride-milled-dehydride (HDH) powders provides a means to control rheology and cost. Binder design needs to balance powder wetting, rheology, green strength, debinding, and contamination concerns. Mixing of the powder and binder at low temperatures prevents oxidation, and mixing under inert gas is generally most beneficial. Solvent debinding, including water and ethanol as solvents, opens the pore structure with minimized contamination. There is contamination from the backbone binders during the last portion of vacuum sweep-gas debinding. Accordingly, experimentation is required to isolate the optimal backbone polymer chemistry and concentration for minimized contamination.

The rheology of highly loaded suspensions depends on the particle size distribution. A broad size distribution is most desirable. However, oxygen concerns lead to removal of the smaller particles, giving a narrow and relatively coarse particle size distribution for titanium. These Ti-MIM feedstocks are different when compared to more traditional systems. The binder tends to be weaker and lower in molecular weight to ease mixing and debinding, but this leads to more molding defects and more required care in green component handling. The powder-binder interface adhesion needs to be improved to reduce separation in molding, a common difficulty with larger particle sizes and low viscosity binders. Efforts with stronger polymers, such as polystyrene, result in increased contamination, so simple wax-like polymers remain the most successful in spite of the low strength and tendency toward separation.

**Figure 3:**
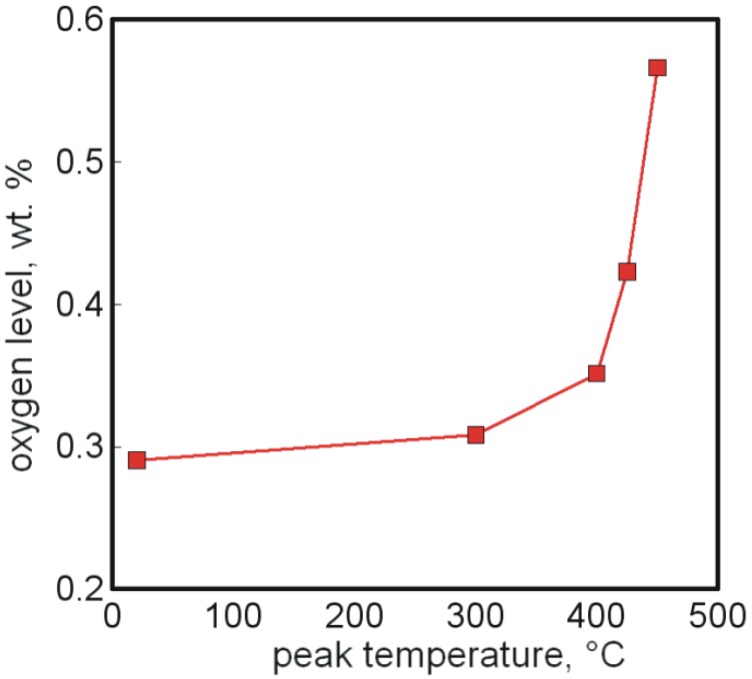
Oxygen content *versus* debinding temperature for titanium powder [136].

Debinding is a sensitive aspect of Ti-MIM and requires two steps; solvent immersion followed by thermal pyrolysis under vacuum using the sweep-gas concept. The peak temperature, hold time, and other parameters are determined using analytical tools, including mass spectroscopy or similar in situ monitors. A good example of the increased oxygen uptake with debinding temperature is shown above in Figure 3 for titanium powder held at various temperatures [136]. Some of the oxygen contamination traces to impurities such as titania (used to catalyze polymerization) carried in by the backbone polymer. Likewise, carbon pick up is common during debinding. In one variant, heating is accelerated during debinding to induce reactions between the titanium and carbon to give TiC, and graphite can be mixed into the feedstock to further enhance titanium carbide formation [64]. This produces a cermet of hard titanium carbides dispersed in a titanium matrix. The composite exhibits high tensile strength, hardness, and wear resistance.

**Figure 4:**
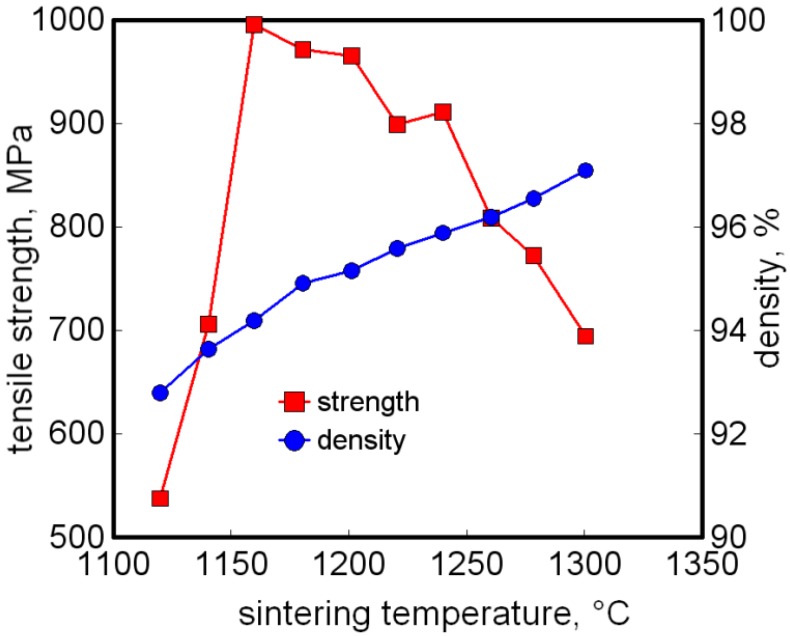
Sintered density and tensile strength for Ti-12Mo *versus* the sintering temperature [64].

The protocol is to isolate a sintering cycle that takes the titanium component to a closed pore condition at about 95% density. Curiously, too high a sintering temperature does damage, with loss of sintered strength due to either gas reactions or microstructure coarsening. This is evident in Figure 4 for Ti-12Mo sintered for 5 h at various temperatures [64]. Subsequently, the component is densified using hot isostatic pressing. The difficult balance between densification in sintering, grain coarsening, gas generation, and contamination is without mathematical analysis, so most trial and error sintering cycles are not optimized.

Now the research turns to Ti-MIM materials for demanding applications and the progress is traced in several recent papers [114,115,116,117,118,119,124,125,128,129,130,131,132,133,134,135,136,137,138,139,140,141,142,143]. Much of this effort is focused on final properties, especially fatigue, as well as oxidation, corrosion, and various biocompatibility attributes. Mechanical properties at elevated temperatures are still largely missing. As case studies emerge to show successful designs, the field will grow toward more widespread acceptance.

But, as research turns to production with mechanical properties rivaling handbook values, the universities efforts are ending. Instead the production facilities are looking to lower cost while qualifying new components, especially in the value-added biomedical and aerospace applications. The early successes help convince designers to use MIM. As a metal, titanium constitutes the largest value in aircrafts (outside the turbine) and second largest value in biomedical. Thus, the challenge is not in developing markets, but in qualifying the Ti-MIM approach.

From these successes, a baseline Ti-MIM process is extracted as given in Table 1. This gives examples of best practices for each of the steps. Variants exist, but this reflects the best current Ti-MIM technology. Changes in the powder or binder result in differences in mixing, impurities, sintering, and other steps. Hence, this is a demonstration of what should work, but is not comprehensive with respect to the many options.

**Table 1:** A baseline Ti-MIM process.

Steps	Key principle	Specific time, temperature, and such
**powder**	deagglomerated spheres	gas or plasma atomized
	typically −325 mesh	30 to 60 μm median particle size
	high tap density	60% to 62% of pycnometer density
	low initial oxygen level	0.15 wt % maximum
	low initial carbon level	0.04 wt % maximum
**binder**	majority low molecular polymer	65% to 75% paraffin wax or polyethylene glycol
	higher molecular weight backbone	15% to 25% polypropylene or ethylene vinyl acetate
	surfactant, lubricant, plasticizer	5% stearic acid
**mixing**	mixing under protective conditions	vacuum or argon cover gas
	room temperature dry mix all ingredients	at 65 vol % solids loading
	heated, high shear mixing	vacuum mix, 30 min at 120 to 185 °C
	temperature and solids loading target viscosity	at 500 s^−1^ of 150 to 250 Pa·s
**molding**	controlled nozzle temperature	120 to 180 °C
	slightly heated mold	30 °C
	injection temperature	160 °C
	injection pressure	30 MPa
	green strength	10 MPa
**debinding**	first stage solvent immersion	60 °C; water for polyethylene glycol, heptane for paraffin wax
	solvent penetration rate	2 mm/h
	second stage thermal debinding	slow heatargon sweep gas in vacuum
	vacuum final step debinding	heat slowly to 450 °C, hold 1 h
	presinter heating for strength	hold near 900 °C for 1 h, vacuum
**sintering**	high temperature sintering	vacuum, refractory metal furnace
	support or substrate materials	yttria or zirconia trays
	peak temperature and time	1250 °C for 120 to 180 min
	sintered density	95% of theoretical, closed pore condition
**densification**	hot isostatic pressing	argon without container
	consolidation conditions	900 °C, 100 MPa, 60 min
**properties**	final density and grain size	99.5% to 100%
	grain size	40 to 100 µm
	microstructure	mixed alpha and beta,10 µm platelets
	final impurity level	0.20% to 0.22% oxygen, 0.04% carbon
	tensile strength	tensile strength 900 MPa
	tensile elongation	12%
	fatigue endurance limit	up to 500 MPa

**Figure 5:**
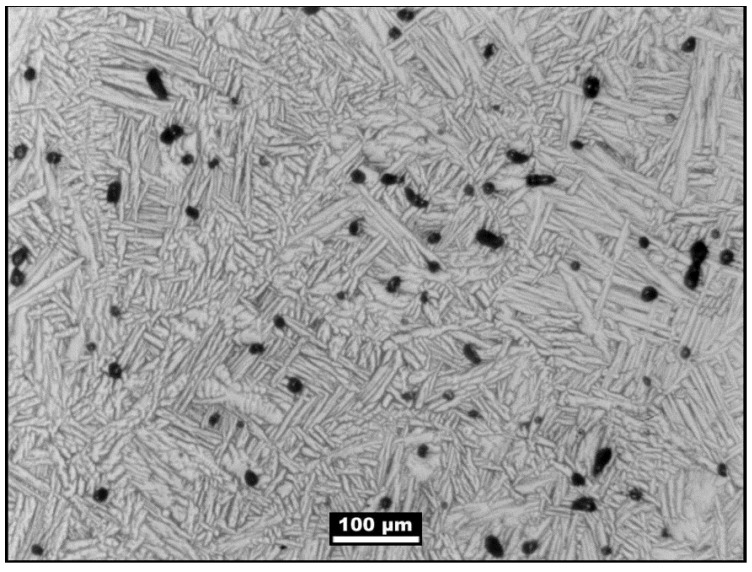
Microstructure for Ti-6Al-4V produced by Ti-MIM prior to hot isostatic pressing.

The need for the post-sintering HIP treatment is evident by the sintered microstructure shown in Figure 5. In this figure the black spots are pores that remain after sintering. Note the large grain size, indicative of considerable coarsening prior to full densification. In this case the sintering temperature resulted in a mixture of two phases, giving a desirable lamellar structure. The grains are over 100 µm in size, the pores are about 10 to 15 µm in diameter, and the lamellar plates are about 5 to 10 µm. Because of the porosity and coarse microstructure, the mechanical properties are typically the same as for cast material.

After the HIP treatment the grain size is similar, but the pores are absent and no artifact of the powder process remains.

## 4. Powder Options

Efforts to lower powder cost have not been fruitful. The different products identified earlier—decorative, mechanical, and life critical—inherently involve different price structures for the more stringent powder attributes. Demanding applications require control over the impurities in the powder. This is because the final product oxygen is higher than the starting powder level. To avoid contamination, the decision is to use a larger particle size as a means to reduce surface area for oxygen contamination. Several powder suppliers produce powders with low interstitial levels at prices priced in the $110 to $220 per kg range, depending on quantity, interstitial level, alloy, and particle size. This is competitive with gas atomized cobalt-chromium MIM powder selling at $160 per kg. On a volume basis, titanium is lower in cost (density of 8.4 g/cm^3^ for cobalt-chromium *versus* 4.5 g/cm^3^ for Ti-6Al-4V). Titanium should displace other metals in biomedical MIM applications, except for stainless steels.

The powder offerings for Ti-MIM fall in the following categories; sponge fines, gas atomization, centrifugal atomization (rotating electrode), plasma atomization, hydride-milled-dehydride (HDH), mechanically spheroidized variants, and novel routes such as electrochemical, calcium reduction, and ultrasonic chemical reduction. Much attention is given to the progress in this area, but generally all routes are finding it difficult to ramp production to meaningful quantities of powder to impact availability issues.

As introduced already, Ti-MIM requires specific powder characteristics:

- Particle size distribution (quantified by the median particle size);
- Particle shape (quantified by the tap density);
- Interstitial level (quantified by oxygen and carbon levels).

**Figure 6:**
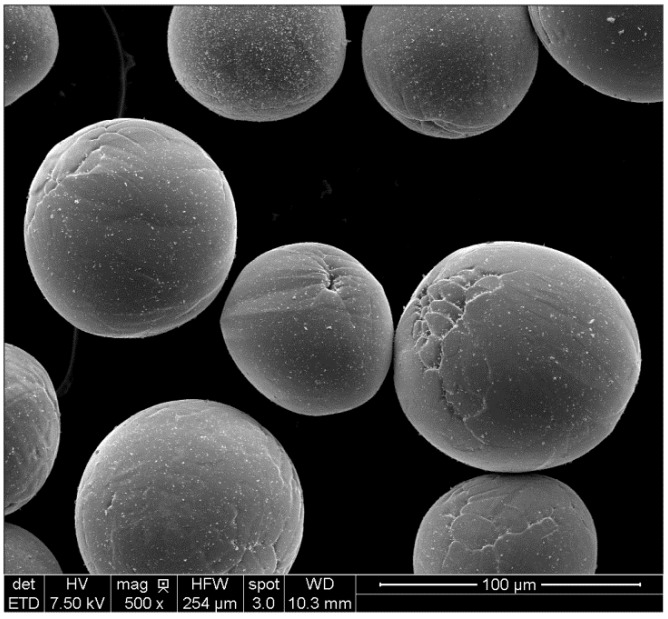
Titanium spherical powder formed using plasma atomization of a hydride-milled-dehydride (HDH) powder.

In addition, not often discussed, there is a requirement for deagglomerated, pore-free powders. Thus, particles without entrapped gas pockets are required, what are termed dense discrete particles. The typical grades used in injection molding are spherical, below 45 µm, and alloyed (Ti-6Al-4V). Figure 6 shows one recent offering, a nearly spherical particle generated by a mixture of hydride-dehydride and plasma atomization. It only suffers from some solidification voids and some inclusions.

Other powders are angular (such as hydride-dehydride) with mechanical treatments to improve packing and flow. Because of difficulty in removing oxygen and carbon, it is necessary to start with impurity levels below the final requirements. Generally, Ti-MIM processing adds from 0.02% to 0.1% interstitials. Final oxygen levels depend on the starting level (powder purity) and change encountered in each PIM step. In some studies, the change in oxygen and carbon from the initial powder to the final product was in excess of 0.15 wt % oxygen and 0.10 wt % carbon. Thus, starting with 0.2 wt % oxygen in the powder gives, with good practices a final oxygen content in the sintered product near 0.3 wt %. Lower levels might be attained by calcium purification of the sintering atmosphere, but this is not documented in any controlled experiments. For a watch case, there is less concern over oxygen, and indeed the higher hardness from an intermediate interstitial content improves wear resistance. On the other hand, medical implant applications clearly demarcate low oxygen and carbon levels; the lowest levels are not attainable via Ti-MIM yet. At least 15 efforts to form low-cost, high purity titanium powders are underway, with names of FCC Cambridge, BHP Billiton, Armstrong, CSIR, and TiRo (CSIRO), but to date significant difficulties arose in scaling to even pilot production, so the Ti-MIM community relies on the standard current powders. Some of the powder offerings for Ti-MIM are summarized in Table 2, giving the typical key powder attributes from the common processes, realizing that over 40 firms are producing powders. Thus, variation is expected between vendors for what is nominally the same composition.

To adjust rheology, HDH powders are mixed with gas atomized powders. The angular HDH powder adds interparticle friction to resist distortion in debinding. Solids loadings up to 72 vol % have been realized when small HDH powders are mixed with larger spherical gas atomized powders. Unfortunately, the HDH powders are higher in impurities, resulting in an increase in final oxygen level as HDH is added to the feedstock. Efforts to balance cost *versus* impurity level include formulation of mixed powders ranging from 100% HDH to 100% gas atomized. In experiments using gas atomized powder with 0.16 wt % oxygen and HDH powder with 0.23 wt % oxygen, after sintering the material derived from pure gas atomized powder produced 550 MPa tensile strength with 23% elongation, while the pure HDH powder resulted in 710 MPa tensile strength and 8% elongation.

**Table 2:** Characteristics of titanium powders used for metal injection molding.

Powder type	Median size, µm	Tap density, % of pycnometer	Oxygen, wt %	Carbon, wt %
sponge fines	38	48	0.35	0.05
hydride-dehydride	38	38	0.25	0.04
titanium hydride	35	40	0.20	0.02
reactive	30	47	0.30	0.10
gas atomized	32	60	0.15	0.03
plasma atomized	60	62	0.15	0.04
rotating electrode	130	72	0.15	0.02

To minimize impurities, powder selection favors a large particle size with less surface area to limit reactions. For example, Chen *et al.* [102] examined different particle sizes of HDH powder using a wax-polymer binder and two-step (solvent and thermal) debinding, followed by vacuum sintering at 1350 °C for 90 min. The smaller particle size required a higher molding pressure, gave slower debinding, and resulted in a higher sintered density. The impurity level after sintering was high. The penalty with a larger powder is less dimensional precision, slower sintering densification, and a rough sintered surface.

A typical compromise for titanium powder is to use −325 mesh (below 45 µm), spherical or tumbled powder customized to give a high tap density. The starting oxygen level is below 0.20 wt % and the carbon level is below 0.05 wt %. One trick is to use hydride powders, where the hydrogen liberated during sintering helps remove volatile impurities, especially those arising from the sintering atmosphere.

Prices for MIM grade titanium powders are highly variable with particle shape, purity, and alloying, and in some special cases reach to $600 per kg. The highest cost corresponds to spherical, prealloyed powder at the lowest interstitial level. The lower cost is from recycle powders, generated by hydriding, milling, and vacuum dehydriding, but starting impurities are high. Titanium powders do not react with oxygen during short duration exposures at room temperature. Once titanium powder ignites the reaction propagates with near-explosive speed reaching up to 100 MPa/s pressure rise. First oxidation occurs near 400 °C, comparable to the peak temperature encountered during debinding burnout. If a powder is sparked, then a fire or explosion is possible. Although not measured, it is anticipated titanium MIM powders will behave similar to aluminum and zirconium powders and will explode when dispersed at concentrations greater than 40 g/m^3^ so this is probably an applicable limit for handling powders in Ti-MIM. The greatest danger is during discharge from mixers, storage containers, or milling devices.

## 5. Components Design Factors

Titanium metal powder injection molding is advancing, but is restricted to about 5% of the metal powder injection molding industry. Many components are formed using Ti-MIM and a few feedstock companies are providing precompounded feedstock.

**Figure 7:**
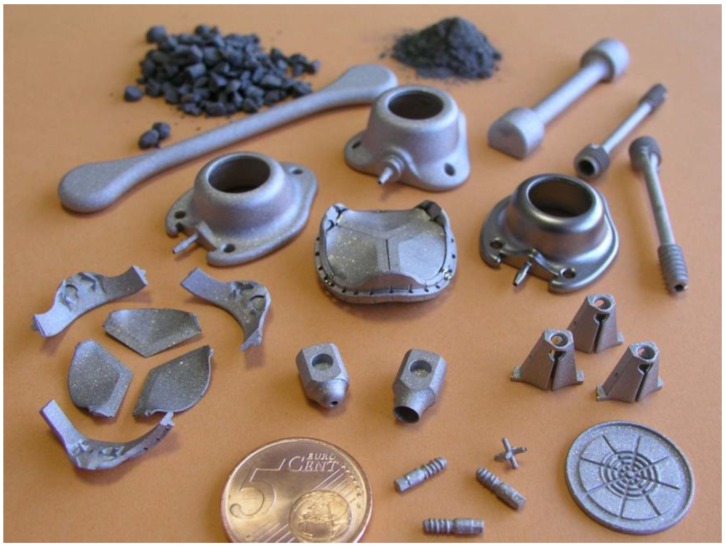
Example Ti-MIM shapes produced by Element 22 GmbH (Kiel, Germany).

Even so, the market for Ti-MIM is small and dwarfed by the success of stainless steels by injection molding, especially for cellular telephone and computer applications. Unfortunately the application data for Ti-MIM is poorly organized and some of the trade associations ignore the activities by about 10% of the industry to develop the practice. Since titanium is significantly higher priced *versus* stainless steel, the 10,000 kg of titanium powder used each year for metal injection molding produces substantial sales. This is because the typical Ti-MIM component sells for $8 each with an average mass of 10 g. In other words, Ti-MIM is probably about $10 million in component sales globally. Some of the early Ti-MIM shapes are shown in Figure 7.

As noted above, a variety of products have been demonstrated using Ti-MIM. The more demanding applications require hot isostatic pressing to ensure full density, which adds to the expense. This is on top of an already high powder cost and cost for contamination control during processing. Unfortunately, the cost advantage is missing for some of the larger scale applications, such as cellular telephone components. Thus, as powder costs decrease there is ample opportunity for expanded applications. So the challenge is to qualify the MIM approach.

The median for MIM production tends toward 10 g and 25 mm maximum size while Ti-MIM is best suited to smaller components [107]. Costs drive Ti-MIM toward low mass components.

There are few reports on Ti-MIM tolerances, but in general the tolerance capabilities are not up to par with other MIM technologies. Whittaker [41] reports a case with gas atomized powder, wax-polymer binder, solvent debinding, and vacuum sintered at 1250 °C for 2 h with a coefficient of variation (standard deviation divided by mean size) of 0.1%. Other reports with less detail on processing claim coefficients of size variation ranging from 0.08% to 0.65% and mass variations from 0.5% to over 1%. Without more details, it would appear Ti-MIM is potentially closing in on the dimensional tolerance capabilities of other MIM approaches, but the sintered surface finish is rougher because of the larger particle size.

## 6. Conclusions

There is nothing routine about Ti-MIM. The field is being advanced by several universities and several powder vendors. The binders for Ti-MIM emphasize lower melting ingredients that are easily extracted in solvents such as water or ethanol. Debinding by first stage solvent immersion reduces contamination. All reports show contamination increasing from powder to product, so the strategy is to start with clean powder and optimize each step in sequence to minimize the contamination increase. Certain binder ingredients seem to add more to the impurity burden, as do different thermal cycles, atmospheres, substrates, peak temperatures, and other variables. The Ti-MIM technology is well-developed and there are several powder vendors supporting the technology. Costs and applications are being rationalized to justify the need for quality powder.
